# Retraction Note: Downregulation of the long noncoding RNA MBNL1-AS1 protects sevoflurane-pretreated mice against ischemia-reperfusion injury by targeting KCNMA1

**DOI:** 10.1038/s12276-021-00698-5

**Published:** 2021-11-08

**Authors:** Xue-Feng Li, Zong-Qiang Wang, Long-Yun Li, Guo-Qing Zhao, Shao-Nan Yu

**Affiliations:** 1 Department of Anesthesiology, China-Japan Union Hospital of Jilin University, Changchun, 130033 PR China; 2 Medical Department, China-Japan Union Hospital of Jilin University, Changchun, 130033 PR China; 3 Department of Radiology, China-Japan Union Hospital of Jilin University, Changchun, 130033 PR China

Retraction to: *Experimental & Molecular Medicine* 10.1038/s12276-018-0133-y, published online 05 September 2018

The authors have retracted this article. After publication they repeated the experiments further increasing the expression of KCNMA1 but found that this did not activate the cGMP PKG signaling pathway. The authors now believe that the mechanism by which the KCNMA1 gene inhibits the cGMP PKG signaling pathway affecting the disease is not one-to-one regulation. All authors agree to this retraction.

